# Prepontine non-giant neurons drive flexible escape behavior in zebrafish

**DOI:** 10.1371/journal.pbio.3000480

**Published:** 2019-10-15

**Authors:** Gregory D. Marquart, Kathryn M. Tabor, Sadie A. Bergeron, Kevin L. Briggman, Harold A. Burgess

**Affiliations:** 1 Division of Developmental Biology, *Eunice Kennedy Shriver* National Institute of Child Health and Human Development, Bethesda, Maryland, United States of America; 2 Neuroscience and Cognitive Science Program, University of Maryland, Maryland, United States of America; 3 Circuit Dynamics and Connectivity Unit, National Institute of Neurological Disorders and Stroke, Bethesda, Maryland, United States of America; University of Sussex, UNITED KINGDOM

## Abstract

Many species execute ballistic escape reactions to avoid imminent danger. Despite fast reaction times, responses are often highly regulated, reflecting a trade-off between costly motor actions and perceived threat level. However, how sensory cues are integrated within premotor escape circuits remains poorly understood. Here, we show that in zebrafish, less precipitous threats elicit a delayed escape, characterized by flexible trajectories, which are driven by a cluster of 38 prepontine neurons that are completely separate from the fast escape pathway. Whereas neurons that initiate rapid escapes receive direct auditory input and drive motor neurons, input and output pathways for delayed escapes are indirect, facilitating integration of cross-modal sensory information. These results show that rapid decision-making in the escape system is enabled by parallel pathways for ballistic responses and flexible delayed actions and defines a neuronal substrate for hierarchical choice in the vertebrate nervous system.

## Abstract 2

Many species execute ballistic escape reactions to avoid imminent danger, but this is not always the best strategy. This study shows that zebrafish rapidly assess the salience of an auditory threat to select either an immediate ballistic response or a delayed and more flexible escape that uses a distinct and newly discovered neural pathway.

## Introduction

Escape behaviors are fast defensive responses to threats that are typically driven by short sensorimotor reflex arcs [1]. However, in humans and other animals, threat imminence strongly influences the selection of defensive strategies [2]. Indeed, some species possess multiple modes of escape, including less powerful responses, characterized by delayed initiation and less-vigorous motor activity [3–5]. Such delayed escape reactions are frequently produced in response to the same stimuli that drive fast escape responses but are preferentially elicited by weaker cues. There has been little work characterizing circuits that mediate delayed escapes [6], precluding analysis of neuronal mechanisms that select and coordinate threat responses.

Escape behavior, triggered by abrupt tactile, auditory, or visual stimuli, has been studied extensively in teleost fish. Central to the escape circuit are the Mauthner cells (M-cells), a bilateral pair of giant reticulospinal neurons that trigger explosive C-start evasive maneuvers with a single action potential [7–9]. However, a second class of escape swim has also been described [10–14]. Zebrafish larvae respond to auditory stimuli with kinematically distinct Mauthner-initiated short-latency C-starts (SLCs) and long-latency C-starts (LLCs) [10,15,16]. Like delayed escapes in other species, LLCs are less vigorous, more variable, and preferentially elicited by weaker stimuli. However, neurons that initiate LLCs have not been described, and it is not known whether LLCs share neuronal pathways with SLCs or why the initiation of LLCs is delayed relative to Mauthner-mediated responses.

To resolve these questions, we conducted an unbiased circuit-breaking screen to identify specific neurons that drive delayed escapes in zebrafish. We discovered a bilateral cluster of approximately 20 neurons per side in the prepontine hindbrain that initiate delayed escapes. Prepontine escape neurons are only active on trials in which larvae initiate a delayed escape but do not project directly to the spinal cord, indicating that they act as premotor neurons. Finally, results from behavioral experiments suggested that delayed escapes facilitate multimodal integration. Our data reveal that rapid behavioral choice is subserved by parallel pathways for ballistic and flexible delayed escapes and provides an identified and experimentally tractable neuronal substrate to study hierarchical decision-making within the vertebrate nervous system.

## Results

**Fig 1:**
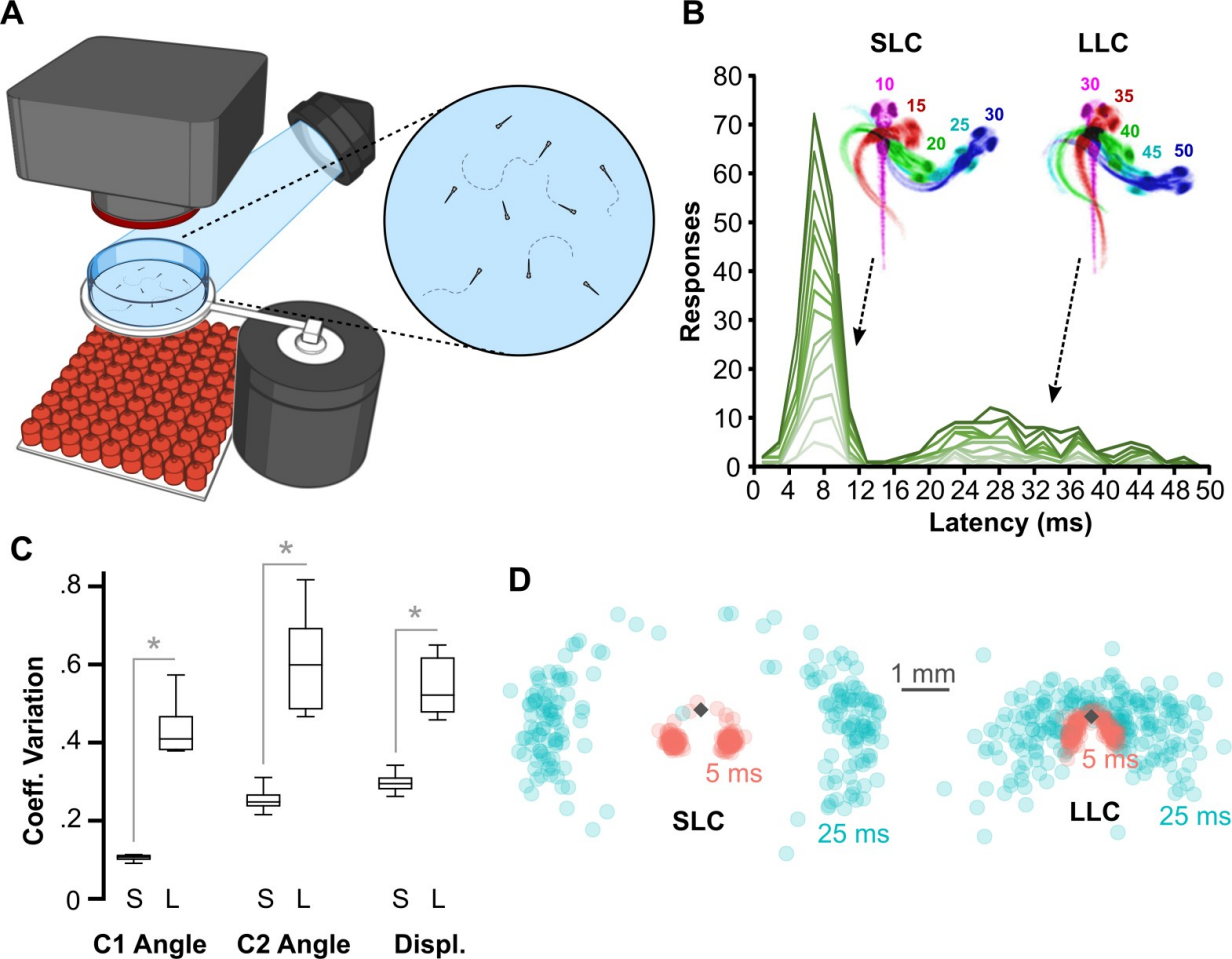
Ballistic and delayed escape reactions performed by larval zebrafish. (A) Schematic of behavioral experiments in free-swimming larvae: groups of 15–20 6-dpf larvae were imaged from above at 1,000 frames per second with a high-speed camera. An infrared LED array below provided illumination. Nondirectional acoustic/vibratory stimuli were delivered to the arena by a minishaker. (B) Frequency histogram of response latencies for individual larvae (*n* = 15). Inset: Time-lapse images of initial C-bend for larvae performing an SLC or an LLC, color-coded by millisecond poststimulus. (C) Coefficient of variation (“Coeff. Variation”) for the initial bend angle (“C1”), counterbend angle (“C2”), and net displacement (“Displ.”) for SLC (“S”) and LLC (“L”) responses. *n* = 16 groups of larvae. *p < 0.001, paired *t* test. (D) Spatial distribution of head locations 5 ms (red) and 25 ms (blue) after escape initiation for SLC (*n* = 199) and LLC (*n* = 220) reactions, relative to initial position (diamond). Underlying numerical data are included in S1 Data. dpf, day post-fertilization; LED, light-emitting diode; LLC, long-latency C-start; SLC, short-latency C-start.

We used high-speed video to analyze escapes triggered by acoustic/vibrational stimuli in free-swimming larvae that were 6 d post-fertilization (dpf) (Fig 1A). Auditory C-start reactions comprise a fast C-bend (C1), counterbend to the other side, and swim bout. As previously described, the distribution of latencies from stimulus onset to C1 initiation was bimodally distributed: larvae initiated an SLC within 12 ms of the stimulus or an LLC 16–50 ms after the stimulus (Fig 1B). We also confirmed that individual larvae executed both SLC and LLC escapes on different trials to stimuli of the same intensity (Fig 1B). Whereas SLCs were highly stereotyped all-or-nothing responses, LLC responses were kinematically variable, showing a significantly greater coefficient of variation for all C1 movement parameters (Figs 1C and S1A), and were less vigorous, resulting in a smaller net displacement (Fig 1D). The relatively long reaction time, high variability, and slower speed of LLCs are features shared with secondary modes of escape in other species [4,5,17].

**Fig 2:**
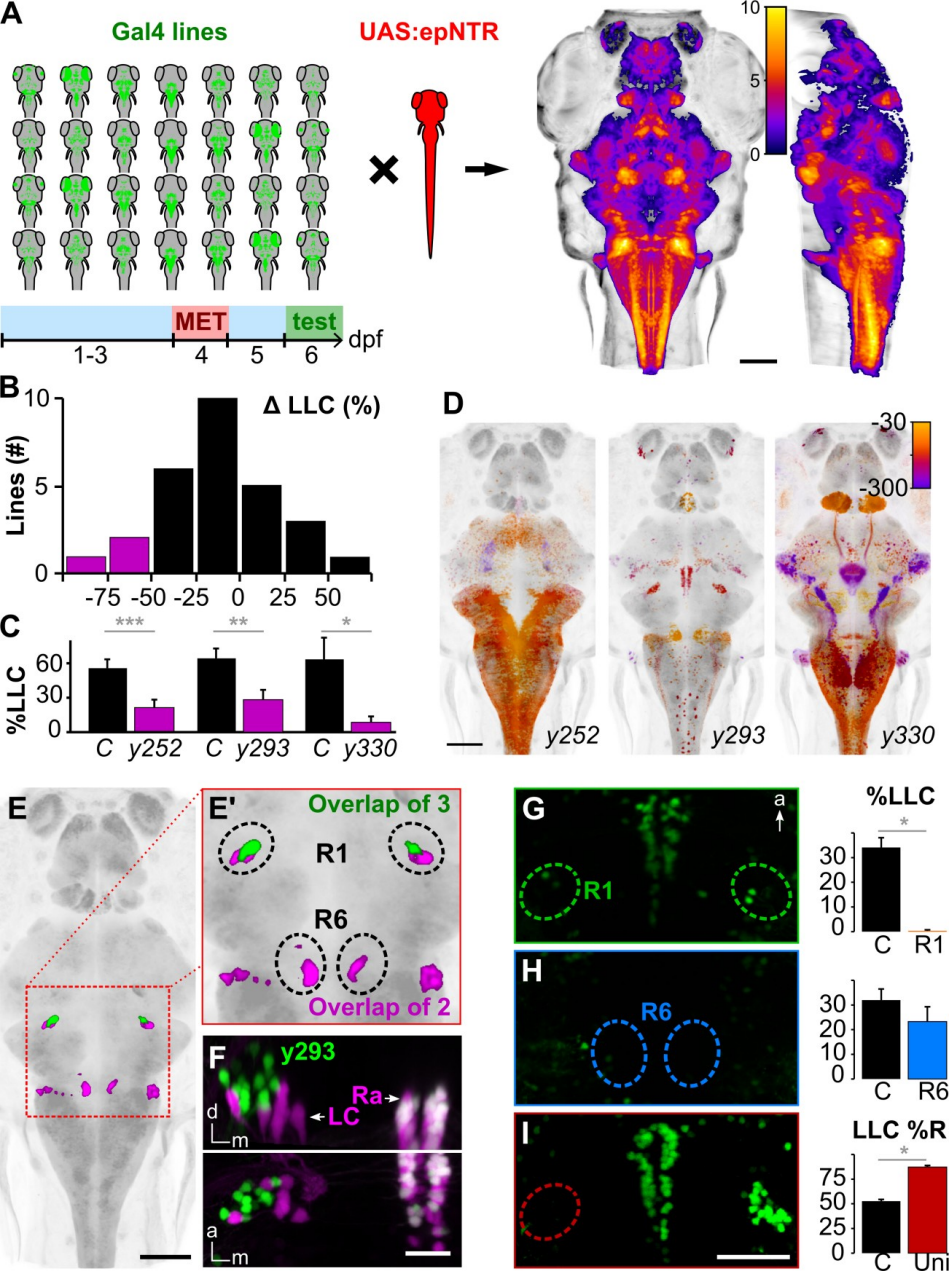
A cluster of neurons in the prepontine hindbrain initiates delayed escapes. (A) Schematic of circuit-breaking screen: 28 Gal4 enhancer trap lines were crossed to *UAS*:*epNTR*, and labeled neurons were ablated and tested for escape behavior. Right: heat maps representing maximum horizontal and sagittal projections of brain coverage (number of lines labeling a given voxel). (B) Histogram of the change in LLC probability following ablation (compared to MET-treated non-epNTR-expressing sibling controls) for each line screened. Magenta: Gal4 lines with a >50% reduction. A minimum of 7 larvae were used per condition per line. %LLC is the percentage of trials on which an LLC was performed as a fraction of trials with no SLC response. (C) LLC probability for lines highlighted in (B). LLC probability after ablation (magenta) and in MET-treated sibling controls (black). *y252-Gal4* (*n* = 22 control, 31 ablated larvae), *y293-Gal4* (*n* = 17, 17), *y330-Gal4* (*n* = 8, 7). ***p < 0.001, **p < 0.01, *p < 0.05, *t* test. (D) Maximum horizontal projections for Gal4 lines with reduced LLC probability after ablation. Expression is color-coded for depth (μm below image top). (E) Expression overlap between *y252-Gal4* and *y293-Gal4* (magenta) and between all 3 lines (green). Boxed area enlarged in (E'). (F) Coronal (top) and dorsal (bottom) projections of confocal substacks through the R1 cluster in *y293-Gal4; UAS*:*Kaede* (green)*; vmat2*:*GFP*^*pku2*^ (magenta) larvae. Prior to imaging, Kaede was photoconverted to red. Arrows indicate LC and Ra labeled by *vmat2*:*GFP*. Both Kaede and GFP are expressed in the raphe (white). (G-H) LLC probability after laser ablation of R1 (G, *n* = 9) and R6 (H, *n* = 16) in *y293-Gal4*. *p < 0.05, *t* test. (I) Percent of LLCs made in a rightward direction after left R1 ablation (“Uni,” *n* = 14) and nonablated controls (*n* = 24). *p < 0.05, *t* test. Scale bars: 100 μm in (A, D, E); 25 μm in (F); 40 μm in (G-I). Underlying numerical data are included in S1 Data, and unprocessed image stacks in (F) are available at https://doi.org/10.5281/zenodo.3382102. a, anterior; d, dorsal; dpf, day post-fertilization; epNTR, engineered nitroreductase variant; GFP, green fluorescence protein; LC, locus coeruleus; LLC, long-latency C-start; m, medial; MET, metronidazole; R1, rhombomere 1; Ra, raphe; SLC, short-latency C-start.

To identify neurons that subserve delayed escapes, we initiated a circuit-breaking screen using a library of Gal4 lines to selectively chemogenetically ablate subsets of neurons using an engineered nitroreductase variant (epNTR, see Methods) before testing escape behavior (Fig 2A). Lines were chosen in the Zebrafish Brain Browser (ZBB) based on three-dimensional (3D) transgene expression patterns to maximize cumulative coverage within the brain [18]. We confirmed 3 lines in which LLC responses were reduced by more than 50% after ablation (*y252-Gal4*, *y293-Gal4*, and *y330-Gal4*; Fig 2B and 2C). Critically, SLC responses were not reduced but in 2 cases were actually increased (*y252-Gal4* and *y293-Gal4*; S1B Fig), indicating that decreases in LLC responsiveness were unlikely to be due to impairments in sensory sensitivity. Additional motor phenotypes differed between the lines, presumably because of the distinct sets of neurons ablated in each (Figs 2D, S1B and S1C). We reasoned that the 3 lines may label a shared population of neurons critical for LLCs and evaluated overlap in co-registered whole-brain images of Gal4 expression [18]. Strikingly, three-way colocalization was restricted to a single area in the prepontine hindbrain: a bilateral region of rhombomere 1 (R1) located dorsolaterally to the locus coeruleus, comprising 19 ± 1.7 neurons per side (mean/s.e.m. for *n* = 10 *y293-Gal4* larvae; Figs 2E, 2F and S2). Neurons in the prepontine cluster were therefore candidates for driving delayed escape behavior.

We next laser ablated prepontine neurons in R1 to test whether they are required for delayed escapes. Focal ablation of the bilateral clusters completely abolished delayed escapes across all stimulus intensities (Fig 2G). In contrast, LLCs were unimpaired in control experiments in which we eliminated a cluster of neurons in R6 that were colabeled by *y252-Gal4* and *y293-Gal4* (Fig 2H, S3A Fig). After unilateral ablation of the prepontine cluster, more than 80% of LLCs were directed toward the intact side (Fig 2I). Prepontine ablations did not affect motor performance but reduced the probability of fast escapes, an effect that is likely to be nonspecific (potentially because of damage to the underlying locus coeruleus) because unilateral lesions did not affect SLC direction (S3B–S3D Fig). Taken together, transgenic and laser ablation experiments reveal that a bilateral cluster of neurons in the prepontine hindbrain are essential for delayed escapes. These neurons are adjacent to the locus coeruleus but not labeled by the monoaminergic marker *vesicular monoamine transporter 2* (*vmat2*) (Fig 2F). The transgene in *y293-Gal4* is integrated in the first intron of *fibronectin type III domain containing 5b* (*fndc5b*) and therefore likely reflects the spatial expression pattern of this gene [18]. In the Allen Mouse Brain Atlas, *Fndc5* is also expressed adjacent to the locus coeruleus, in the vestibular nuclei [19,20], a region previously implicated in driving vestibular startle responses in mammals (S4 Fig) [21,22]. These and other similarities (see Discussion) suggest that prepontine escape neurons in fish may be homologous to the mammalian superior vestibular nucleus (SUV).

**Fig 3:**
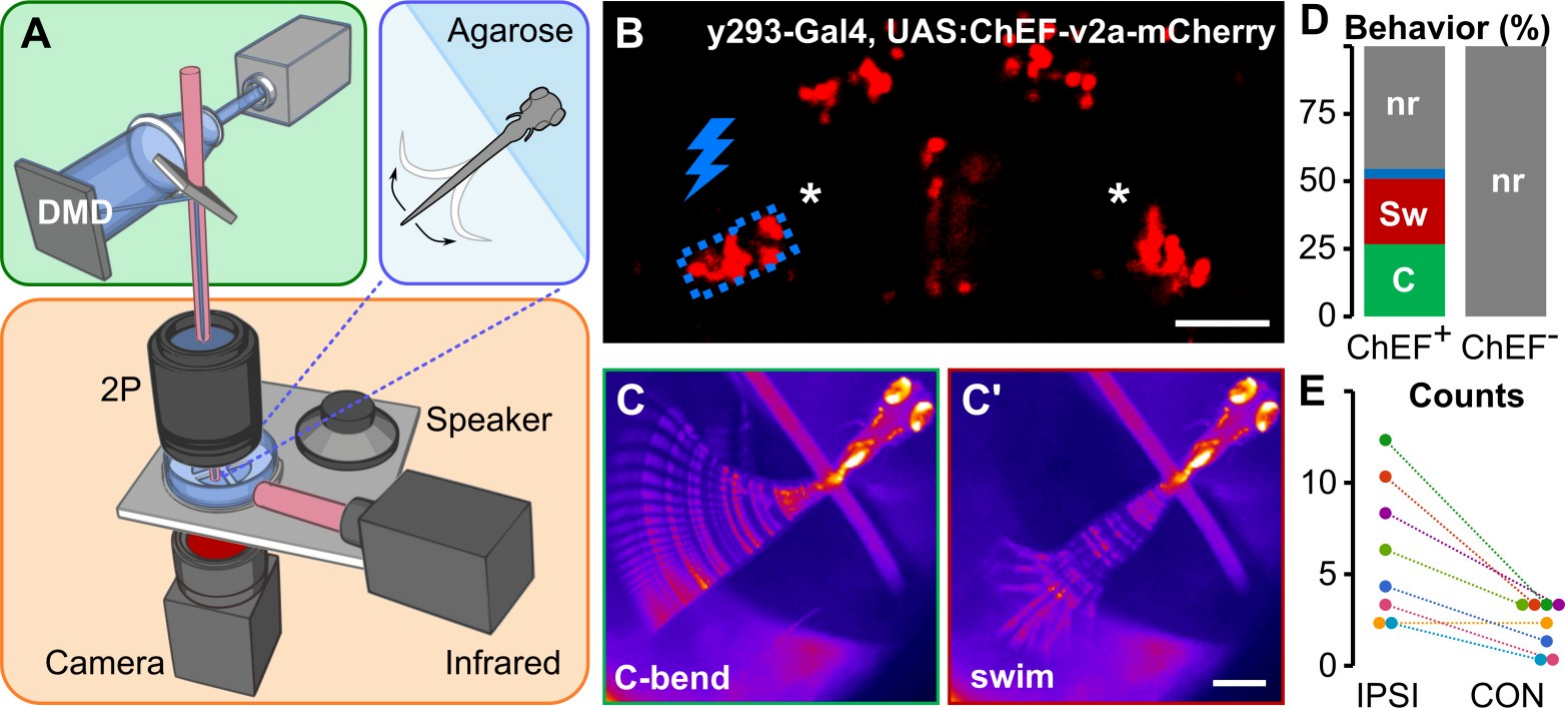
Optogenetic prepontine neuron stimulation elicits C-start behavior. (A) Schematic of optogenetic stimulation and two-photon calcium imaging: a DMD was used to spatially restrict 460-nm LED excitation (green box) within the brain of head-embedded larvae (blue box) mounted on a stage with a speaker for acoustic/vibratory stimulation, an infrared light source for tail illumination, and a high-speed camera for behavioral readout (orange box). (B) Two-photon optical section of mCherry expression in *y293-Gal4*, *UAS*:*ChEF-2a-mCherry* larva with the area around one prepontine cluster (asterisks) stimulated by the DMD outlined in blue. Scale bar 40 μm. (C) C-start and swim-like (C') behaviors elicited by unilateral optogenetic stimulation of prepontine neurons in *y293-Gal4*, *UAS*:*ChEF*-positive larvae. Scale bars 500 μm. (D) Percent of behaviors elicited by illumination of larvae expressing ChEF (ChEF^+^; 229 trials, *n* = 8 larvae) and nonexpressing sibling controls (ChEF^−^; 63 trials, *n* = 7 larvae). C-start-like responses (“C,” green), swim-like bouts (“Sw,” red), other responses (blue), nr (gray). (E) Number of C-start responses made ipsilateral (“IPSI”) and contralateral (“CON”) to the side of optogenetic stimulation, color-coded for each of the 8 larvae tested. χ^2^ = 15.25, *p < 0.001. Underlying numerical data are included in S1 Data. DMD, digital mirror device; LED, light-emitting diode; mCherry, monomeric Cherry; nr, no response.

Prepontine neurons might directly initiate escape reactions or regulate the responsiveness of another pathway. To test whether prepontine neuron activation drives escape behavior, we expressed the channelrhodopsin variant ChEF in *y293-Gal4* neurons and selectively stimulated prepontine neurons in head-embedded larvae using a digital mirror device (DMD) (Fig 3A and 3B)[23]. Control ChEF-negative larvae did not respond to light-emitting diode (LED) illumination. Unilateral illumination of ChEF-positive neurons elicited behavioral responses in 54.6% of trials, of which half were initiated with a large-angle tail flexion similar to C-start responses in free-swimming fish (Fig 3C and 3D, and S1 Video). C-start-like responses triggered by unilateral optogenetic activation were primarily initiated to the ipsilateral side (Fig 3E). These results confirm that prepontine neurons drive escape-like behavior and support the finding from unilateral lesion experiments that neurons in each hemisphere predominantly, although not exclusively, drive ipsilateral responses.

**Fig 4:**
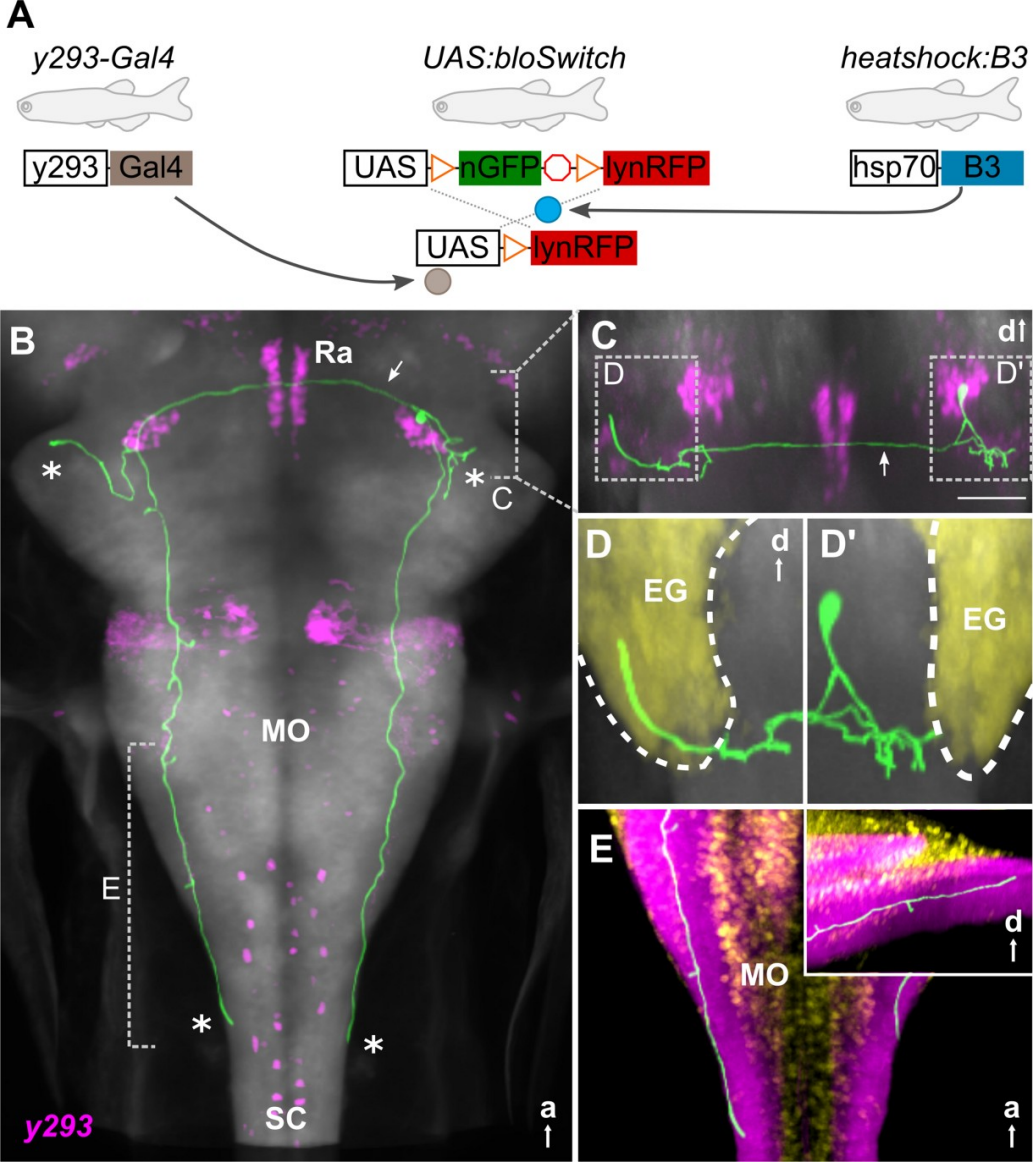
Prepontine escape neurons project reciprocally to the caudal hindbrain and cerebellum. (A) Schematic of 3 transgene system used for B3-recombinase–based neuronal tracing. (B-E) Representative traced neuron in 6-dpf larva (green, for others, see S5 Fig), registered to ZBB atlas [18]. Background is *elavl3*:*Cer* (gray). (B) Horizontal maximum whole-brain projection of a reconstructed neuron from *y293-Gal4* (ZBB, magenta). Asterisks: projections of the 4 primary neurites. Arrow: commissural projection. Dashed lines indicate views in (C) and (E). (C) Coronal substack projection from the area indicated in (B). Arrow: commissural projection. Scale bar 50 μm. Views in (D) outlined. (D) Coronal projections of neurites extending into the ipsilateral (D') and contralateral (D) EG (yellow). (E) Dorsal projection through the caudal medulla lateral neuropil area (ZBB *anti-zrf2*, purple). Cellular regions labeled by *Tg(elavl3*:*nls-mCar)y517* (ZBB, yellow). Inset: sagittal view of same region. a, anterior; d, dorsal; EG, eminentia granularis; hsp70, heat-shock protein 70; lynRFP, membrane-tagged red fluorescent protein; MO, medulla oblongata; nGFP, nuclear-localized green fluorescent protein; Ra, raphe; SC, spinal cord; ZBB, Zebrafish Brain Browser.

Rapid reaction times for M-cell–initiated escapes are achieved through a short sensorimotor pathway, use of electrical synapses, and the large caliber of the Mauthner axon [24]. Thus, during fast escapes, the VIIIth nerve directly activates Mauthner neurons, which form monosynaptic contacts with motor neurons throughout the contralateral spinal cord [25,26]. As a step toward characterizing the delayed escape pathway, we reconstructed prepontine *y293* neurons. For tracing, we sparsely labeled neurons by crossing *y293-Gal4* to a heat shock–inducible B3 recombinase and an upstream activating sequence (UAS) reporter with B3 recombinase “blown-out” (blo) recognition sites (Fig 4A) [27]. B3 is relatively inefficient in larval zebrafish, allowing heat-shock conditions to be titrated to achieve stochastic expression of membrane-tagged red fluorescent protein (RFP) from the UAS:bloSwitch reporters [28]. We imaged 20 prepontine neurons and manually reconstructed 5 to visualize their morphology, revealing bilateral terminations in the cerebellar eminentia granularis (EG) and the caudal hindbrain (Figs 4B and S5A). A single neurite from each neuron projected ventrally then bifurcated into lateral and medial branches (Fig 4C). The lateral branch terminated nearby, arborizing in or below the EG (Fig 4D). The medial branch split again: one fork extended through a dense neuropil area to the caudal hindbrain (Fig 4E), and the other crossed the midline (through the raphe nucleus) to the bifurcation zone of neurites from the contralateral prepontine cluster (Fig 4B and 4C, arrows). Here, as on the ipsilateral side, the process split, arborizing within the EG (Fig 4D) and extending to the caudal hindbrain (Fig 4E). This quadripartite morphology was shared by all 20 neurons imaged, with the only salient differences being (1) the caudal extent of hindbrain projections (S5B Fig) and (2) whether neurites projected bilaterally into the EG (S5C Fig). Neurites were not apposed to VIIIth nerve projections (S5D and S5E Fig). Moreover, although *y293* neurons form presynaptic terminals in a neuropil zone at the same rostro-caudal level as the anterior-most population of motor neurons (S5F and S5G Fig), they do not project further into the spinal cord and therefore cannot directly activate motor neurons throughout the length of the spinal cord to drive a C-start response. Thus, unlike the pathway for fast escapes, in which only 3 synapses are interposed between hair cells and motor neurons, prepontine escape neurons are not directly connected to either sensory input or all necessary motor output neurons, which likely contributes to the longer latency of the response.

**Fig 5:**
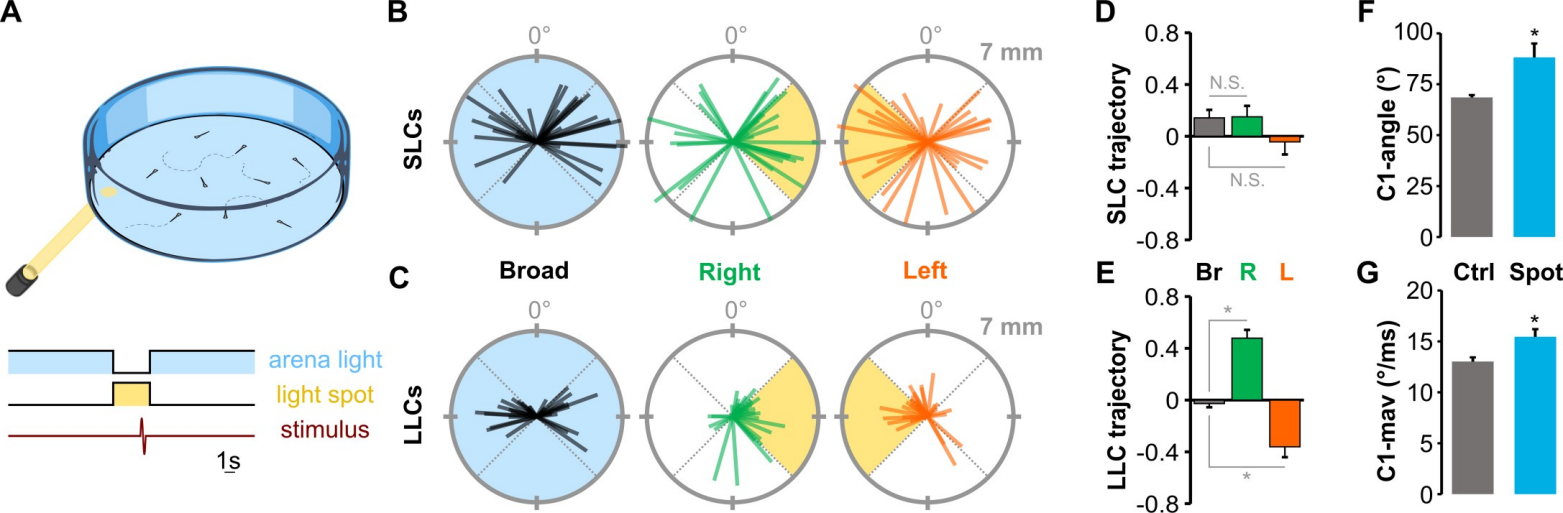
Delayed escape trajectories are guided by visual information. (A) Schematic of experiment measuring escape direction under broad-field illumination or in darkness with only a light spot illuminated. Scale bar 1 s. (B-C) Thirty representative SLC (B) and LLC (C) escape trajectories of larvae to a nondirectional acoustic/vibratory stimulus when under broad-field illumination (“Broad”) or when oriented to the left or to the right of a light spot. Escape direction is plotted radially and net displacement axially. (D-E) Mean direction choice (−1 all left; +1 all right) for SLC (D) and LLC (E) responses under broad-field illumination (“Br”; SLC, *n* = 367 responses; LLC, *n* = 372) or when the light-spot was to the left (“L”; SLC, *n* = 131 responses; LLC, *n* = 257) or to the right of the larva (“R”; SLC *n* = 143; LLC, *n* = 283). *p < 0.001, *t* test. (F-G) Mean initial bend angle (F) and maximum angular velocity (G) for LLCs performed under broad-field illumination (gray) or during directionalized responses with a light spot. *p < 0.01, *t* test. Underlying numerical data are included in S1 Data. Ctrl, control; LLC, long-latency C-start; N.S., not significant; SLC, short-latency C-start.

We reasoned that the extended pathway and greater reaction time for delayed escapes may provide an opportunity to integrate additional information from the environment to guide LLC trajectories. Because zebrafish larvae are strongly attracted to light, we combined a light spot with an acoustic stimulus and tested escape trajectories (Fig 5A). In this paradigm, nondirectional broad-field illumination on control trials was replaced with a localized light spot several seconds before delivery of a nondirectional acoustic stimulus. Whereas SLC trajectories were similar during broad-field illumination and during light-spot exposure, LLC trajectories were preferentially performed toward the spot (Fig 5B–5E). Moreover, body curvature and angular velocity during the initial C-bend were increased during directionalized delayed escapes, whereas other kinematic parameters were unchanged (Figs 5F, 5G and S6A). Directionalized responses were absent in *atonal bHLH transcription factor 7* (*atoh7*) mutants, which lack retinofugal projections, confirming that retinal signaling is responsible for guiding delayed escape trajectories (S6B Fig). Thus, external visual cues strongly influence LLC but not SLC escape trajectory in larvae, consistent with the idea that the longer latency of delayed escapes provides additional time for integration of sensory information into computations that guide path selection.

**Fig 6:**
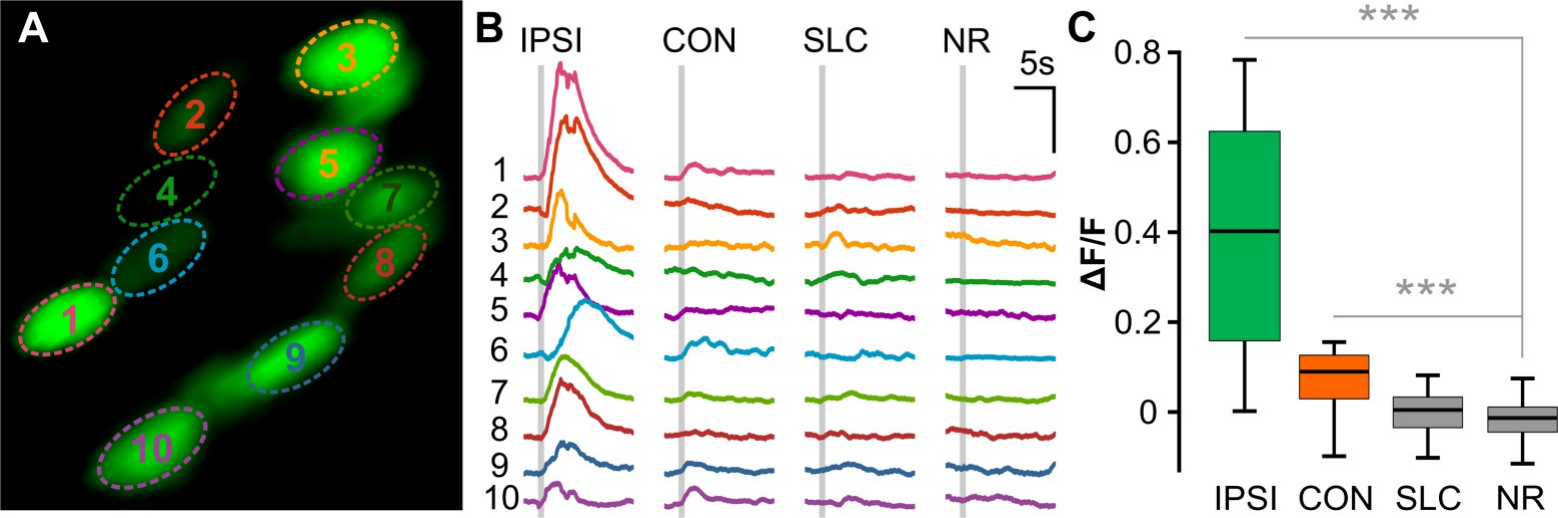
Prepontine neurons are active during ipsilateral delayed escapes. (A) Two-photon optical section of nuclear-localized GCaMP6s-positive prepontine neurons in *y293-Gal4* with ROIs shown in (B) indicated. (B) Representative GCaMP6s traces for ROIs in (A) for ipsilateral LLCs (“IPSI”), contralateral LLCs (“CON”), SLCs, and NR trials. Each vertical set of GCaMP6s traces are from the same trial. Gray bar: acoustic stimulus. Scale bar 1 ΔF/F, 5 s. (C) Change in GCaMP6s fluorescence (ΔF/F) across response types: ipsilateral LLCs, contralateral LLCs, SLCs, and NR trials (31 neurons, *n* = 3 larvae). ***p < 0.001, *t* test. Underlying numerical data are included in S1 Data. LLC, long-latency C-start; NR, no response; ROI, region of interest; SLC, short-latency C-start.

**Fig 7:**
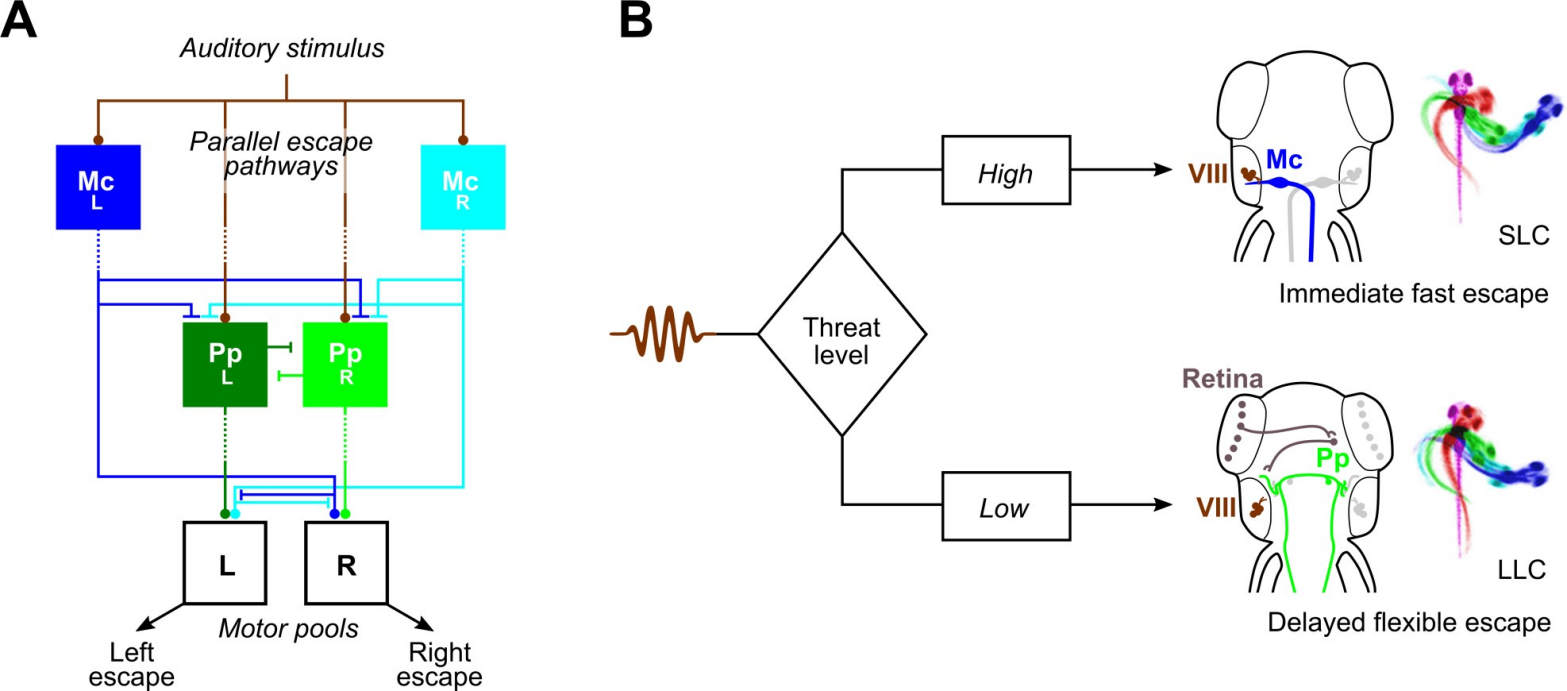
Escape pathways in zebrafish. (A) Parallel sensory pathways transmit acoustic information to M-cells (“Mc_L_” and “Mc_R_”) and prepontine escape neurons (“Pp_L_” and “Pp_R_”). Decision-making is based on reaction time: VIIIth nerve activation of M-cells is direct, whereas prepontine neurons receive auditory information only via an indirect pathway, allowing active M-cells to prevent the initiation of delayed escapes. Additionally, Mauthner cells inhibit prepontine escape neurons, likely through an indirect pathway. Dashed lines indicate indirect connections. (B) Anatomy corresponding to the model in (A). Auditory signals from the statoacoustic ganglion (VIII, brown) excite M-cells (“Mc,” blue) directly (top) or prepontine escape neurons (“Pp,” green) indirectly (bottom). M-cells receive predominantly ipsilateral inputs and project commissurally to drive fast escapes, whereas prepontine escape neurons project both ipsi- and contralaterally and may drive escape in either direction, although predominantly ipsilaterally. LLC, long-latency C-start; M-cell, Mauthner cell; SLC, short-latency C-start.

To test whether prepontine escape neurons integrate sensory information, we performed two-photon calcium imaging of nuclear-localized GCaMP6s in head-embedded *y293-Gal4* larvae. In parallel, we monitored tail movements in order to correlate activity with behavior. We simultaneously recorded from multiple prepontine *y293-Gal4*, *UAS*:*GCaMP6s* neurons during presentation of an auditory stimulus and then grouped responses based on the behavioral outcome (Fig 6A). Mean SLC responsiveness was 20.4% ± 1.5%; however, unexpectedly, LLC responses were only elicited on 1.1% of trials in embedded larvae. The low rate of delayed escapes in immobilized larvae precluded us from testing the effect of directionalized light stimuli. Nevertheless, on trials with a delayed escape, prepontine neurons on the same side as the initial C-bend showed a significant increase in mean activity (Fig 6B and 6C). Activity was less elevated, but also above baseline, on trials with a delayed escape on the contralateral side. However, neurons were completely inactive on trials in which the acoustic stimulus failed to elicit a reaction, demonstrating that the prepontine clusters are not sensory interneurons but motor-associated neurons whose activity correlates most strongly with ipsilateral delayed escape reactions. Strikingly, prepontine escape neurons were also silent on trials in which the larva performed a fast escape. This suggests that fast escape and delayed escape pathways are not coactive and suggests that Mauthner-mediated fast escape responses suppress the delayed escape pathway. We propose that the auditory stimulus recruits independent pathways for escape and that the faster reaction time of the M-cell pathway shuts down the delayed escape circuit, preventing transmission of potentially conflicting motor commands (Fig 7).

## Discussion

Rapid escape responses in many species are mediated by giant fiber neurons, providing a conspicuous entry point into the underlying circuit [29]. In contrast, neuronal pathways for alternate modes of escape have not been well characterized. Here, we reveal a population of premotor neurons that initiate delayed escape behavior in larval zebrafish: a bilateral cluster of approximately 20 neurons per side adjacent to the locus coeruleus in the prepontine hindbrain. Although prepontine escape neurons project bilaterally to the caudal medulla oblongata, calcium imaging, lesion, and optogenetic activation experiments all indicate that these neurons predominantly initiate ipsilateral escapes. Delayed escape trajectories are strongly biased by visual cues from the environment, suggesting that these responses represent a more “deliberative” mode of escape, potentially allowing larvae to better evade predators or obstacles.

The pathway we describe for auditory-induced delayed escapes is not similar to previously described escape circuits in zebrafish. Rapid C-start escapes to head-touch stimuli are mediated by the reticulospinal neurons MiD2cm and MiD3cm [30], and slow-velocity looming stimuli trigger non-Mauthner escapes, potentially via a set of reticulospinal neurons that show stimulus-correlated activity [6]. However, unlike reticulospinal neurons, which traverse the medial and lateral longitudinal fasciculi to contact targets in the spinal cord, prepontine escape neurons project through lateral fiber tracts and terminate in a dense neuropil zone in the caudal hindbrain and must therefore drive at least the majority of spinal cord motor neurons indirectly. Similarly, unlike the Mauthner neurons, which receive monosynaptic auditory input from the VIIIth nerve, prepontine escape neurons must receive polysynaptic inputs, potentially within the EG, a region known to receive sensory input and where prepontine neurite morphology resembles dendritic arborizations [31]. A third difference is the mechanism for selecting escape direction. Feedforward inhibitory signals help to select activation of a single M-cell [14]; however, acoustic stimuli often activate both M-cells, and downstream mechanisms prevent simultaneous bilateral activation of motor pools [32]. In contrast, prepontine neurons show much greater activity on the side ipsilateral to the escape direction. Commissural processes project reciprocally to the contralateral nucleus, raising the possibility that lateral inhibition ensures unilateral activation and initiation of an escape to one side.

Although Mauthner-initiated escape responses in adult fish are biased by visual cues, our data indicated that at larval stages, only delayed escape trajectories were biased by visual information [33]. In mammals, distinct neural pathways mediate active flight from imminent threats and "slow strategic escape" from distant dangers [34]. LLCs, although slow compared to Mauthner-initiated responses, are nevertheless rapid defensive reactions rather than a cognitive avoidance behavior. However, our data suggest that delayed escapes may provide a window for cross-modal integration and computation of an optimal escape trajectory to evade threats or obstacles. In addition, less vigorous, long-latency escapes may allow animals to calibrate the cost of behavioral responses to perceived threat [6]. Consistent with this idea, LLCs are preferentially evoked by weak acoustic stimuli and match response vigor and speed to stimulus intensity [10,16]. In some circumstances, the predictable path trajectories of fast escapes are susceptible to exploitation [35]. Indeed, many prey species show “protean behavior,” exhibiting intrinsically erratic or variable responses to confuse predators [36]. For zebrafish, the presence of alternate modes of escape and the intrinsic variability of delayed escape behavior may reduce the predictability of escape trajectories. These roles are not exclusive: faced with a less precipitous threat, larvae may compute an optimal escape trajectory that is also energetically favorable and more flexible than M-cell–driven fast escape reactions.

Delayed escape neurons are located in an unannotated area of the prepontine hindbrain between the locus coeruleus and the cerebellum. The *y293-Gal4* line is an enhancer trap for *fndc5b*, which is expressed in a topographically similar area in mice that is annotated as the vestibular nucleus (VN). This is striking because the VN drives startle responses to abrupt vestibular stimuli in mammals [21,22]. The precise pathway for vestibular startle has not been characterized but, like the prepontine delayed escape pathway, is independent of the system that drives startle responses to acoustic or somatosensory stimuli [37]. Among components of the VN, the SUV is most similar to prepontine escape neurons: of R1 origin [38] and, at least in frog, comprising neurons with commissural and possibly cerebellar projections [39]. Future experiments with molecular markers will be required to provide definitive evidence, but based on position, projections, and function, we provisionally propose that prepontine escape neurons reside in the zebrafish homolog of the mammalian SUV and may represent an evolutionarily ancient secondary pathway for rapid defensive responses to threats sensed via acoustic or vibrational cues.

Why are multiple prepontine escape neurons active during LLC responses? A single M-cell spike defines the latency and direction of an SLC; this spike, however, does not encode the entire movement trajectory but acts together with parallel descending motor pathways [40]. Although it is possible that parallel descending pathways also encode LLC escape parameters, we speculate that coactive prepontine escape neurons collectively encode primary LLC escape parameters such as the speed and duration of the initial C-bend. In mammals, the strength of startle responses reflects the extent of recruitment and firing rate of reticulospinal neurons in the pontine nucleus caudalis [41,42]. Similarly, the graded vigor of delayed escapes according to stimulus intensity may be explained by the number and/or firing rate of prepontine neurons that respond to an auditory stimulus.

The M-cell ventral dendrite receives polysynaptic visual information via the optic tectum [43,44]; however, visual inputs may need to be acute “looming” stimuli to be integrated into M-cell-mediated escape decisions [6]. In our experiments using a static light spot, only delayed escapes integrated directional visual information into trajectory decisions. One possibility is that the integration of visual information requires the additional time provided by delayed escape. If so, this does not reflect transmission time from the retina, as the directional visual information was available for several seconds before the auditory stimulus. Rather, delayed responses may reflect the complexity of multimodal integration, providing sufficient time for the network of prepontine neurons to incorporate visual and auditory information into a trajectory decision. How different visual and auditory streams are segregated between and integrated within M-cell and prepontine escape circuits provides a tractable neuronal substrate for future work on decision-making, multimodal integration, and circuit organization at the cellular level.

Although we do not yet have a comprehensive picture of how short- and long-latency escape circuits interact, we can predict features of their organization (Fig 7). Prepontine neuron activity during SLC escapes would likely interfere with either the counterbend or swim phase of the response, making it essential to prevent delayed escapes during the performance of SLC responses. Indeed, M-cell-mediated escapes override competing motor commands [45]. The large caliber of the Mauthner axon ensures that its motor commands reach the spinal cord before other active descending fibers, allowing inhibition of competing signals [46]. However, M-cell inhibition of delayed escapes does not rely on spinal inhibition, as our calcium imaging experiments revealed that prepontine neurons do not fire during M-cell-initiated SLC responses. M-cell firing must therefore, likely indirectly, inhibit prepontine escape neurons or their inputs. This hierarchical structure may be a general feature within decision-making circuits in which first-to-fire ethologically critical responses suppress slower, less urgent behaviors.

The M-cell system has given us one of the most complete pictures of neural circuit function underlying behavior in vertebrates and remains an important model for understanding how motor decisions are computed at the cellular level [14,43,47]. However, decision-making in a system in which a single neuron can induce key components of a motor behavior may be different than decisions that are implemented by groups of coactive neurons. Our identification of prepontine escape neurons provides an opportunity to explore how collective decisions are implemented at the circuit level and compare how strikingly similar motor programs are initiated either by a population or by a single discrete cell. Finally, prepontine escape neurons may serve as an archetype for identifying delayed escape neurons in other model systems.

## Methods

### Ethics statement

All in vivo experimental procedures were conducted according to National Institutes of Health guidelines for animal research and were approved by the NICHD Institutional Animal Care and Use Committee (#18–007).

### Animal husbandry

Wild-type zebrafish (*Danio rerio*) and Gal4 enhancer trap and transgenic lines used in this study were maintained in a Tüpfel long fin (TL) strain background. Experiments were performed on larvae in the first 8 dpf, before sex differentiation. Embryos were raised in E3 medium supplemented with 1.5 mM HEPES (pH 7.3) (E3h) at 28°C on a 14 h:10 h light:dark cycle with medium changes at least every 2 d unless otherwise described.

### Mutant and transgenic lines

Images throughout were registered to the ZBB to enable comparison with other markers [18]. Gal4 lines used for the circuit-breaking screen were previously described [18,48] and maintained using *Tg(UAS-E1b*:*Kaede)s1999t* (*UAS*:*Kaede*) [49]. We applied the “subdivide pattern” command in the ZBB to the *huc*:*Cer* pattern to select an initial set of 16 lines with good global coverage and then manually selected additional lines to fill in less well represented areas. For chemogenetic ablation experiments, these lines were crossed to nitroreductase lines *Tg(UAS-E1b*:*BGi-epNTR-TagRFPT-oPre)y268Tg* or *Tg(UAS*:*epNTR-TagRFPT-utr*.*zb3)y362Tg* [18,50]. Gal4 lines used in chemogenetic ablation experiments were brain-specific, containing neuronal-restrictive silencing elements [48], or were crossed to the *y362Tg* line that includes a synthetic 3′ UTR designed to suppress non-neural expression [18]. Gal4 lines are available from the Zebrafish International Resource Center (ZIRC; http://zebrafish.org). *UAS*:*bloswitch* and *hsp70l*:*B3* lines were used for *y293Et* neuron tracing [28]. *Atoh7*^*sa16352*^ mutants were acquired from the ZIRC [51]. The locus coeruleus was visualized using *Et(gata2a*:*EGFP)pku2* (*vmat2*:*GFP*) [52]. Images of *TgBAC(chata*:*Gal4-vp16)mpn202* (*chata-Gal4*) were as published [53], registered to ZBB. Other lines used were *Tg(mnx1*:*GFP)ml2* [54] and *Tg(UAS-E1b*:*synaptophysin-TagRFPT)y261* [55].

### Imaging

Embryos were raised in E3h media containing 300 μM N-Phenylthiourea (PTU) starting at 8–22 hpf to suppress melanophore formation with PTU changed at least every 48 h. For imaging at 6 dpf, larvae were anesthetized in 0.24 mg/mL tricaine methanesulfonate (MS-222) for 3 min and mounted in 2.5% low-melting-point agarose in 3D-printed plastic inserts (ABS from Stratasys or clear resin from FormLabs) within #1.5 thickness (0.17 ± 0.005 mm) cover glass-bottom cell-culture chambers (Lab-Tek II 155379). An inverted laser-scanning confocal microscope (Leica TCS SP5 II) equipped with an automated stage, and a 25x/0.95 NA apochromatic water immersion lens (Leica # 11506340) was used to acquire confocal stacks. For labeling individual neurons, *y293-Gal4; UAS*:*bloSwitch* fish were crossed to *hsp70l*:*B3*. Sparse labeling was achieved by a 25–35-min heat shock at 37°C at 3 dpf to induce B3 recombinase expression. Larvae were then raised under standard conditions and imaged at 6 dpf. Neurons were traced in Imaris 8.4.2, exported as TIFs, and converted to NIFTI for alignment with ANTs to *y293-Gal4* as a reference [56]. To photoconvert Kaede from green to red in selected neurons, we scanned with a 405-nm laser at 30 mW for 90 s.

### Genetic and laser ablations

For genetic ablations, we used an engineered variant (epNTR) of the bacterial nitroreductase gene, which converts a cell-permeable substrate (metronidazole) into a cell-impermeable cytotoxin [50,57]. Gal4 enhancer trap lines were crossed to UAS:epNTR-TagRFPT and embryos screened for red fluorescence. Nonfluorescent embryos were used as controls. At 4 dpf, larvae were exposed to 10 mM metronidazole for 24 h, given 24 h to recover, and then tested for escape behavior at 6 dpf. For laser excisions, subsets of Kaede-positive neurons were selectively ablated at 4 dpf in *y293-Gal4*;*UAS*:*Kaede* larvae raised in PTU. Laser excisions were performed on an upright laser-scanning confocal microscope (Leica TCS SP5 II) equipped with a multiphoton laser (SpectraPhysics MaiTai DeepSee), automated stage, and 20x/1.00 NA apochromatic water-dipping lens (Leica # 11507701). Larvae to be ablated as well as controls were mounted in 2.5% low-melting-point agarose on #1.5 thickness cover glass, which was then inverted for the upright microscope. A 488-nm argon laser line was used to visualize target cells and confirm ablation, whereas the multiphoton laser tuned to 800 nm was used for selective laser excision. The laser was pulsed for 5–1,000 ms at approximately 2.4 W until cell integrity was compromised. Following ablation, larvae were raised in E3h until behavioral testing at 6 dpf. Successful ablations were then confirmed by confocal microscopy—only larvae with 3 cells or fewer remaining on either side were analyzed.

### Free-swimming behavior

For light-spot experiments, TL larvae were tested in groups of 15–20 within a 33 × 33-mm corral, which kept larvae in view of a high-speed camera. Larvae were illuminated from above at about 80 μW/cm^2^ (arena light) and by an infrared LED array for imaging purposes from below. During light-spot trials, the arena light was replaced for 3.5 s with a light spot of about 8 μW/cm^2^ and an approximately 6-mm diameter focused from below. Three seconds after appearance of the light spot, larvae were exposed to an acoustic/vibratory stimulus. Aside from the switching of illumination, control trials to quantify baseline responses were performed under the same conditions. Control and light-spot trials were pseudorandomly presented 20–40 times each at 15-s intervals. The change in illumination elicited O-bends only within the first second, and not at 3 s when the acoustic stimulus was provided. For chemogenetic ablation experiments, larvae were tested individually in 9.7 × 9.7-mm wells of a 3 × 3 grid illuminated by an LED array at approximately 500 μW/cm^2^ from below. Different stimulus intensities were presented 20 times each in a pseudorandom sequence at 15-s intervals to minimize habituation. Genetically ablated larvae and metronidazole-treated controls were tested in alternation.

Auditory stimuli consisted of sinusoidal waveforms of 21–36 dB, of 2-ms duration, and nominally 250 or 1,000 Hz, though the acoustic/vibrational stimuli as delivered are intrinsically broadband. Stimuli were delivered with an electrodynamic exciter (Type 4810 Mini-shaker; Brüel & Kjær) controlled by a digital–analog data acquisition card (PCI-6221; National Instruments). Behavioral responses of larvae were recorded at 1,000 frames/s with a high-speed camera (DRS Lightning RDT/1 or RL Redlake MotionPro; DEL Imaging) fitted with a 50-mm macro lens (EX DG Macro, Sigma). For light-spot experiments requiring infrared illumination, cameras were additionally fitted with an infrared filter (R72, Hoya Filters). Recorded trial bouts were 120 ms in length with acoustic/vibratory stimuli delivered at 30 ms, except for laser excisions of *y293-Gal4* larvae, for which we analyzed 200 ms to ensure that LLCs were absent and not merely delayed.

Behavioral responses were analyzed with Flote software [10]. As ongoing locomotion differentially influences SLC and LLC probability, larvae were only included if they were motionless in the 30 ms prior to acoustic stimulus. Startle responses were considered SLCs if they occurred within 12 ms of stimulus delivery. LLC responsiveness was calculated as the mean proportion of larvae responding with an LLC, as a fraction of all larvae still stationary after the time period during which SLC responses occur [10]. This adjustment is made because SLC production precludes the production of LLCs. For light-spot experiments, larvae were pooled by quadrant based on their initial orientation to the light spot. For behavioral analysis following PTU treatment, some low-contrast larvae were unable to be automatically tracked with Flote, and behaviors were manually assessed with the scorer blinded to the identity of ablated versus control conditions.

### Calcium imaging and optogenetic activation

For calcium imaging or optogenetic stimulation, *y293-Gal4* embryos were injected at the one-cell stage with tol1 mRNA and a plasmid containing either *UAS*:*BGi-nls-GCaMP6s*.*zf2-v2a-nls-dsRed*.*zf1-afp* or *UAS*:*BGi-ChEF-v2a-mCherry-afp*, respectively [28], screened for red fluorescence, and raised in PTU in the dark. At 6 dpf, GCaMP6s- or ChEF-positive larvae were embedded in 3.5% low-melting-point agarose in E3h in a petri dish with agarose cut away from the tail caudal to the swim bladder to allow for tail movement and behavioral readout of acoustic or optogenetic stimulation. Larvae were then placed on a custom 3D-printed stage with temperature maintained at 28°C by a ring-shaped Peltier device. To track tail movements, larvae were illuminated using a 980-nm LED and imaged from below at 100 or 200 frames per second (fps) using an infrared CCD camera (Pike F-032C IRF, Allied Vision Technologies). Tail movements were acquired and tracked using a custom Matlab script. Each larva was tested with a mean of 8 trials at intervals of 5–10 min. A trial comprised 4 stimuli at interstimulus intervals of 60–90 s. Only larvae that performed both SLCs and LLCs were included for analysis.

GCaMP6s- or ChEF-positive neurons were imaged on a custom-built multiphoton microscope with a 20x/0.90 NA water-dipping lens (Olympus) and a Ti-Sapphire laser (Coherent Chameleon Vision-S) tuned to 950 nm and controlled in Matlab (Mathworks) by ScanImage [58]. For calcium imaging, GCaMP6s signals from single planes through left or right prepontine neurons were imaged at 1.95–13.95 fps. For optogenetic stimulation, two-photon images of prepontine neurons in *y293-Gal4* were converted into binary ROIs and projected back onto the larval zebrafish brain by a 460-nm LED (Prizmatic) and digital micromirror device (DLi4130, Digital Light Innovations) for durations of 10 or 100 ms controlled by Clampex (pCLAMP 10.4, Molecular Devices). Optogenetic-stimulation ROIs covered an area of 50 × 25 μm centered over a prepontine neuron cluster in one hemisphere. Spatial specificity of the setup was previously assessed by photoconversion of Kaede in a targeted area (Fig 3A in [28]). GCaMP6s ΔF/F was quantified in nuclear ROIs drawn in Fiji with frames representing approximately 1 s averaged and compared 2.72 s after acoustic stimulation to those 1 s immediately prior to stimulation. Trials with spontaneous tail movement within 100 ms prior to acoustic stimulation were excluded from analysis.

### Statistics

Analysis was performed with IDL (Harris), R (http://www.R-project.org/), and Gnumeric (http://projects.gnome.org/gnumeric/). All *t* tests are two-sided, independent samples (except where paired-sample tests were used, as noted). No animals were excluded from analysis except for calcium imaging, for which only SLC- and LLC-responsive animals were analyzed or for which laser ablations were incomplete. Graphs and text report means and standard errors. Box plots show median and quartiles; whiskers show 10%–90%. Bar plots show mean and standard error. *N* reported in figure legends indicates biological replicates (number of individual larvae or independent groups of larvae).

## Supporting information

S1 Data Main data. Excel spreadsheet containing numerical data underlying main figures.
(XLSX)

S2 Data Supporting data. Excel spreadsheet containing numerical data underlying supporting figures.
(XLSX)

S1 Fig Additional motor phenotypes after chemogenetic neuronal ablation. (A) Comparison of initial bend angle (C1-angle), counterbend angle (C2-angle), and displacement for SLC and LLC responses (same larvae as in Fig 1C). Probability and kinematic measures for SLC (B) and LLC (C) responses after ablating neurons labeled in *y252-Gal4*, *y293-Gal4*, and *y330-Gal4*. Significant ANOVAs are indicated at the top of each graph, after Bonferroni correction for the 16 comparisons, and *p < 0.05 for post hoc *t* test compared to nonablated metronidazole-treated non-epNTR expressing sibling controls. Underlying numerical data are included in S2 Data. epNTR, engineered nitroreductase variant; LLC, long-latency C-start; Mav, maximum angular velocity; SLC, short-latency C-start.
(TIF)

S2 Fig Prepontine escape neurons are located between the LC and CE. (A-B) Dorsal (A) and parasagittal (B) projections from ZBB of *y293-Gal4-*, *y264-Gal4-*, and *chata-Gal4*-labeled neurons in R1–4. Prepontine neurons labeled by *y293-Gal4* are located in R1, in contrast to the anterior (“a”) and posterior trigeminal motor nuclei labeled by *chata-Gal4* located in R2 and R3, respectively, and the Mc in R4 labeled by *y264-Gal4*. (C) Coronal projection of *y293-Gal4* prepontine neurons situated between the LC and the CE. CE, cerebellum; d, dorsal; LC, locus coeruleus; m, medial; Mc, Mauthner cell; R1–4, rhombomeres 1–4; Ra, raphe; ZBB, Zebrafish Brain Browser.
(TIF)

S3 Fig Additional phenotypes after laser ablation of neurons in R1 and R6. (A-B) LLC (A) and SLC (B) responsiveness after R1 ablation (*n* = 9, green), R6 ablation (*n* = 16 blue), and unablated sibling controls (*n* = 27, black). Significant effects of R1 ablations on LLC and SLC probability; ANOVA F1,102 = 23.37, *p* < 0.001 and F1,102 = 21.79, *p* < 0.001, respectively. (C) SLC kinematic measurements after bilateral R1 laser ablation (*n*'s as above). No significant differences (*t* test). (D) SLC directionality (%Right: percent of SLC responses initiated to the right) after unilateral (left) R1 laser ablation. *n* = 14 (ablated) and 24 (control). Underlying numerical data are included in S2 Data. LLC, long-latency C-start; Mav, maximum angular velocity; R1, rhombomere 1; SLC, short-latency C-start
(TIF)

S4 Fig Prepontine escape neurons in fish are similar to the mouse SUV. (A) Bottom: coronal projection through zebrafish rhombomere 1 (slice 512–517 from ZBB) with nuclear labeling on the left (*elavl3*:*nls-mCar* in purple) and neuroanatomic segmentation on the right (magenta, OT; yellow, CE; pink, medulla oblongata; gray, neuropil). Top: coronal projection of the outlined region showing *y293-Gal4* (green, prepontine neurons), *neurod*:*GFP* (yellow, CE) [59], and *TH-Gal4* (magenta, LC) [60]. (B) Bottom: mouse P56 coronal section (slice 111 from the AMBA) with Nissl-staining on the left (purple) and neuroanatomic segmentation on the right (magenta, superior colliculus; yellow, CE; pink, medulla oblongata; gray, fiber tracts). Top: AMBA in situ hybridization images for *TH* (B) and *Fndc5* (B'). *Image credit*: *Allen Institute*, *modified from Allen Developing Mouse Brain Atlas*. AMBA, Allen Mouse Brain Atlas; CE, cerebellum; *Fndc5*, *fibronectin type III domain containing 5*; HB, hindbrain; LAV, lateral vestibular nucleus; LC, locus coeruleus; MV, medial vestibular nucleus; OT, optic tectum; P56, postnatal day 56; Pp, prepontine area; SUV, superior vestibular nucleus; *TH*, *tyrosine hydroxylase*; ZBB, Zebrafish Brain Browser.
(TIF)

S5 Fig Traces of individual prepontine escape neurons. (A) Dorsal standard-deviation projections of 5 traced prepontine escape neurons with *elavl3*:*Cer* as a reference (gray). (A’) Overlay of co-registered neurons in (A) showing conserved quadripartite morphology (asterisks). Dotted lines indicate areas expanded in (B) and (C). (B) Enlargements of hindbrain from A’ with arrowheads indicating neuron terminals. (C) Enlargements of lateral rhombomere 1 from A’ with arrowheads marking termini in the cerebellar EG. (D) Horizontal projection of confocal stack including prepontine escape neuron cell bodies (“Pp,” *y293-Gal4; UAS*:*KaedeR*, red) after selective photoconversion of Kaede to red and SAG axon rostral termini (*y256-Gal4;UAS*:*KaedeG*, green). (E) Projection of a reconstructed neuron (green) registered to ZBB, with the *y256-Gal4* pattern (magenta) that labels the SAG and its projections. (F-G) Horizontal projection (F) and zoom (G) of confocal stack through the caudal hindbrain and anterior spinal cord of a *y293-Gal4*, *UAS*:*synaptophysin-TagRFPT*, *mnx1-GFP* larva. Scale bars: 50 μm in (A) and (A'); 20 μm in (D-G). EG, eminentia granularis; SAG, statoacoustic ganglion; ZBB, Zebrafish Brain Browser.
(TIF)

S6 Fig Kinematic measures for LLCs directionalized by a light spot. (A) Kinematic parameters for acoustically evoked LLCs performed under broad-field illumination (black) or in the presence of a light spot (blue). *n* = 29 groups of larvae. (B) Percent of LLCs in a rightward direction in the presence of a light spot for *atoh7* mutant larvae and siblings. ***p < 0.001, *p < 0.05, *n* = 5 plates each atoh7−/− and siblings. Underlying numerical data are included in S2 Data. *atoh7*, *atonal bHLH transcription factor 7*; C1, initial C-start bend; C2, counterbend; LLC, long-latency C-start.
(TIF)

S1 Video Optogenetic stimulation of *y293-Gal4* prepontine neurons. Representative optogenetic trials from 3 ChEF-positive and a ChEF-negative control larvae (bottom right) showing behavioral results to DMD illumination and optogenetic stimulation of prepontine neurons in *y293-Gal4*. Light stimulation (460 nm) for 10 or 100 ms is indicated by a square in the top right corner of each subframe with timestamp at the bottom right. DMD, digital mirror device.
(AVI)

## Abbreviations

3D: three-dimensional
*atoh7*: *atonal bHLH transcription factor 7*
blo: blown-out
DMD: digital mirror device
dpf: days post-fertilization
EG: eminentia granularis
epNTR: engineered nitroreductase variant
*fndc5b*: *fibronectin type III domain containing 5b*
LED: light-emitting diode
LLC: long-latency C-start
M-cell: Mauthner cell
nGFP: nuclear-localized green fluorescent protein
N.S.: not significant
R1: rhombomere 1
RFP: red fluorescent protein
SLC: short-latency C-start
SUV: superior vestibular nucleus
UAS: upstream activating sequence
*vmat2*: *vesicular monoamine transporter 2*
VN: vestibular nucleus
ZBB: Zebrafish Brain Browser
