# Supplementary figures and images for: Prepontine non-giant neurons drive flexible escape behavior in zebrafish
Source: PLoS Biol. 2019 Oct 15;17(10):e3000480. doi: 10.1371/journal.pbio.3000480 (PMC6793939; doi:10.1371/journal.pbio.3000480)

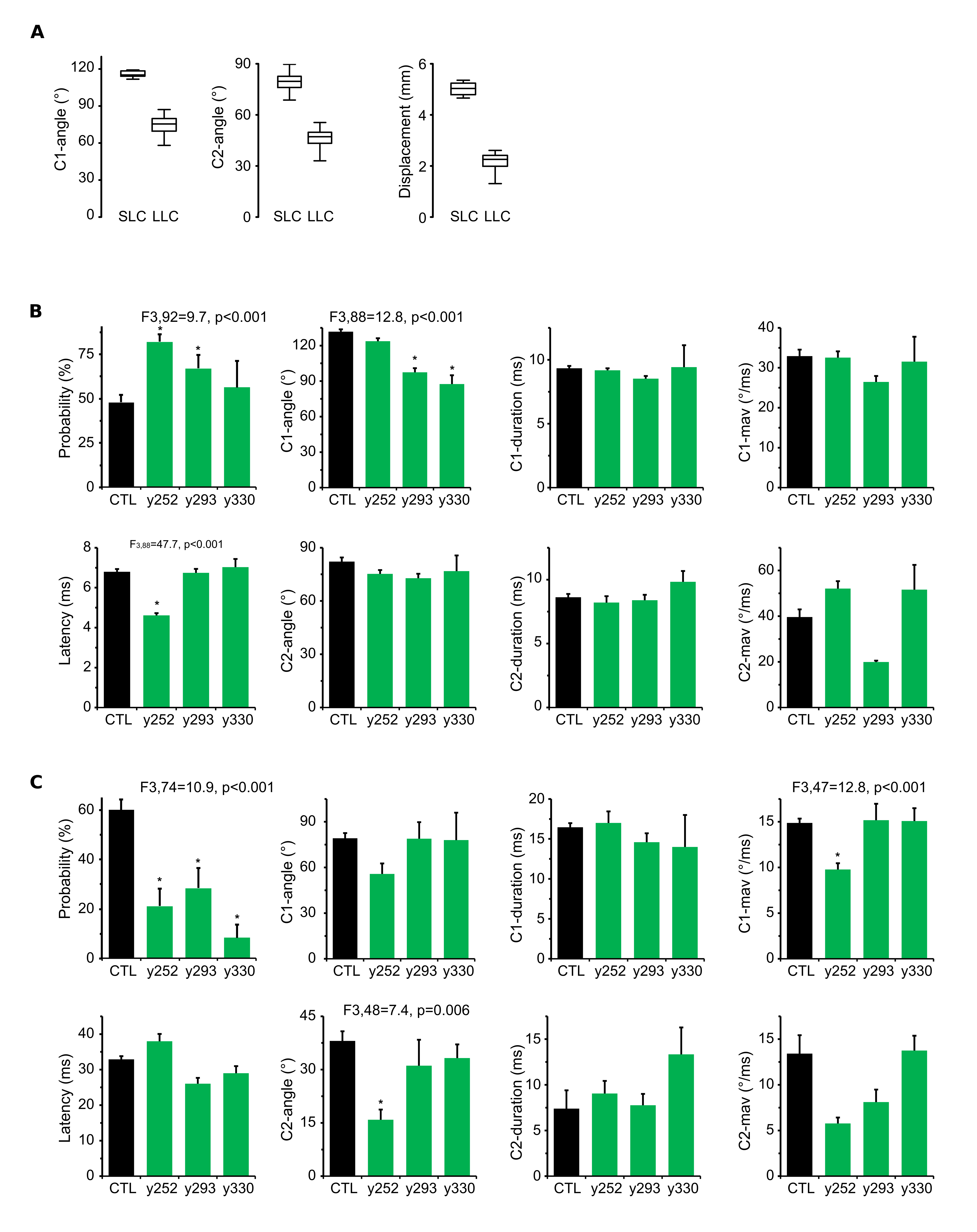

Supplement: S1 Fig — (A) Comparison of initial bend angle (C1-angle), counterbend angle (C2-angle), and displacement for SLC and LLC responses (same larvae as in Fig 1C). Probability and kinematic measures for SLC (B) and LLC (C) responses after ablating neurons labeled in y252-Gal4, y293-Gal4, and y330-Gal4. Significant ANOVAs are indicated at the top of each graph, after Bonferroni correction for the 16 comparisons, and *p < 0.05 for post hoc t test compared to nonablated metronidazole-treated non-epNTR expressing sibling controls. Underlying numerical data are included in S2 Data. epNTR, engineered nitroreductase variant; LLC, long-latency C-start; Mav, maximum angular velocity; SLC, short-latency C-start. (TIF) [file pbio.3000480.s003.tif]

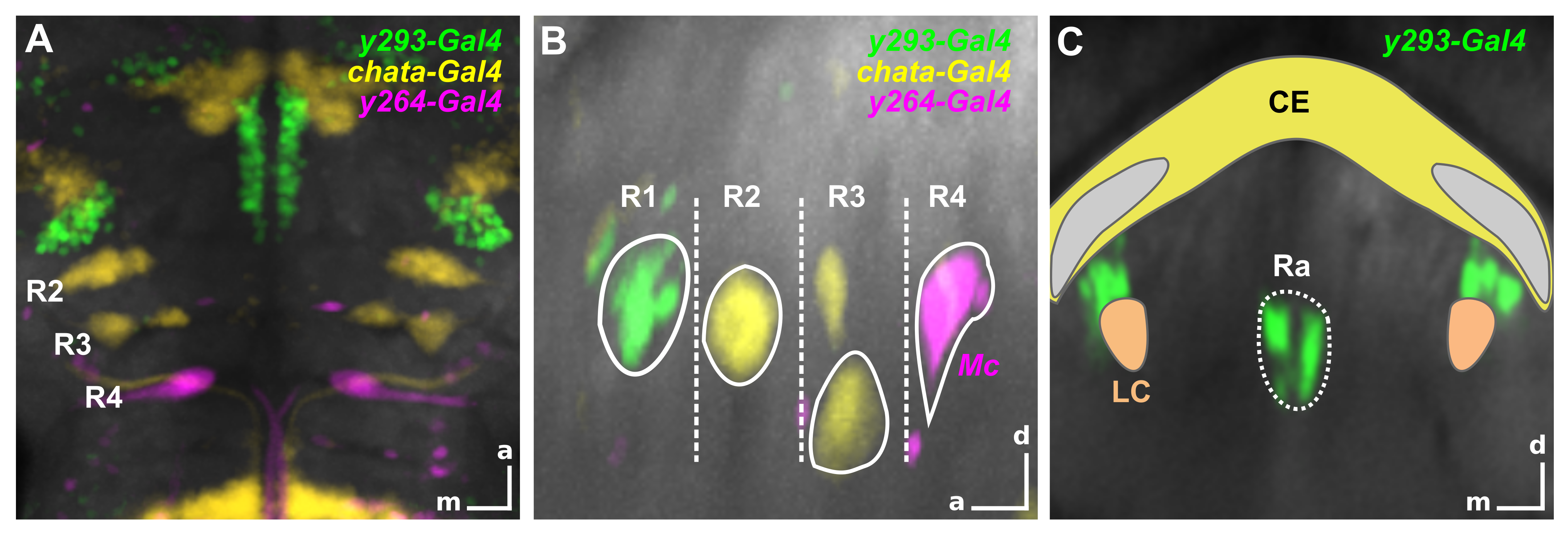

Supplement: S2 Fig — (A-B) Dorsal (A) and parasagittal (B) projections from ZBB of y293-Gal4-, y264-Gal4-, and chata-Gal4-labeled neurons in R1–4. Prepontine neurons labeled by y293-Gal4 are located in R1, in contrast to the anterior (“a”) and posterior trigeminal motor nuclei labeled by chata-Gal4 located in R2 and R3, respectively, and the Mc in R4 labeled by y264-Gal4. (C) Coronal projection of y293-Gal4 prepontine neurons situated between the LC and the CE. CE, cerebellum; d, dorsal; LC, locus coeruleus; m, medial; Mc, Mauthner cell; R1–4, rhombomeres 1–4; Ra, raphe; ZBB, Zebrafish Brain Browser. (TIF) [file pbio.3000480.s004.tif]

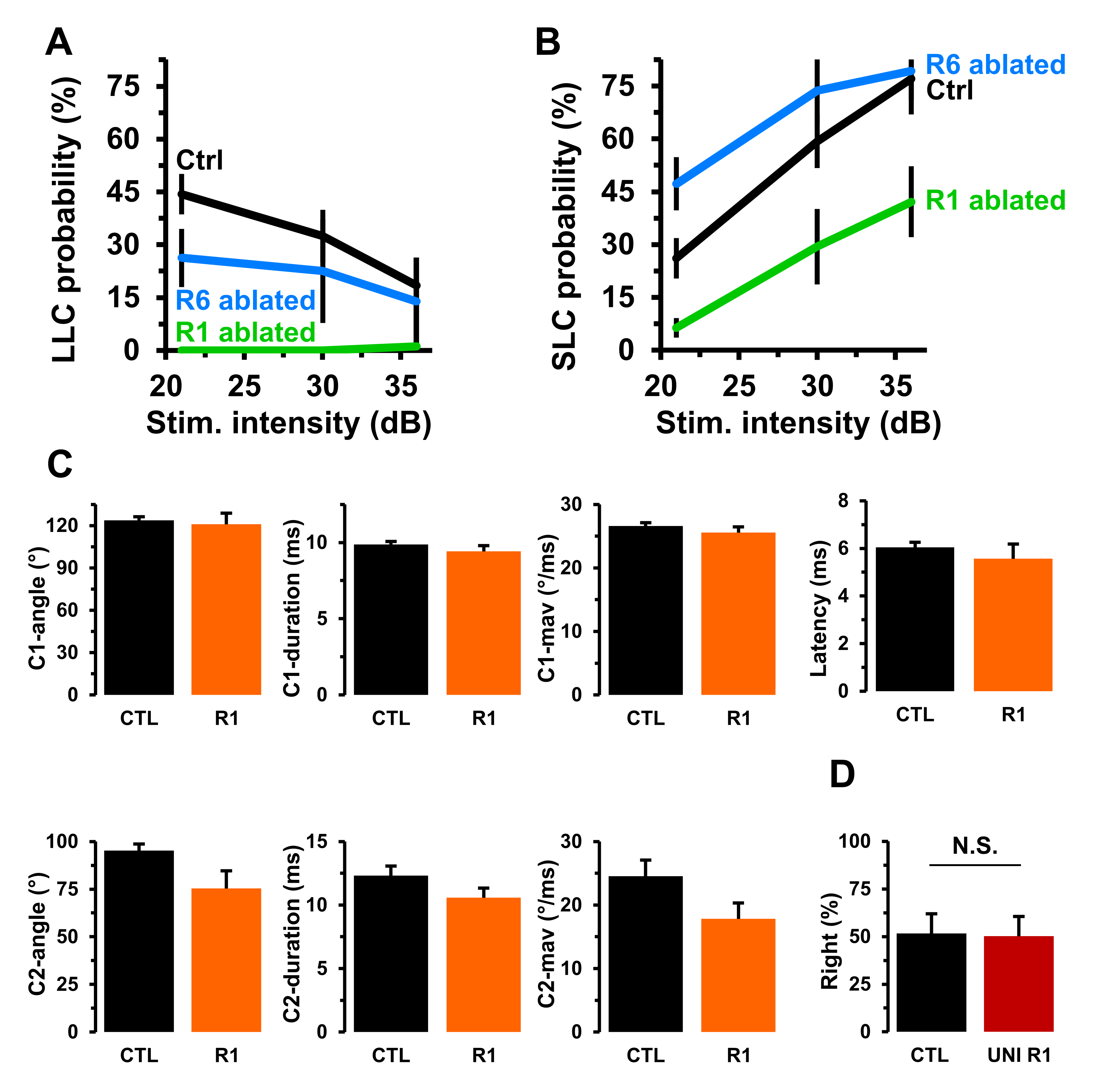

Supplement: S3 Fig — (A-B) LLC (A) and SLC (B) responsiveness after R1 ablation (n = 9, green), R6 ablation (n = 16 blue), and unablated sibling controls (n = 27, black). Significant effects of R1 ablations on LLC and SLC probability; ANOVA F1,102 = 23.37, p < 0.001 and F1,102 = 21.79, p < 0.001, respectively. (C) SLC kinematic measurements after bilateral R1 laser ablation (n's as above). No significant differences (t test). (D) SLC directionality (%Right: percent of SLC responses initiated to the right) after unilateral (left) R1 laser ablation. n = 14 (ablated) and 24 (control). Underlying numerical data are included in S2 Data. LLC, long-latency C-start; Mav, maximum angular velocity; R1, rhombomere 1; SLC, short-latency C-start (TIF) [file pbio.3000480.s005.tif]

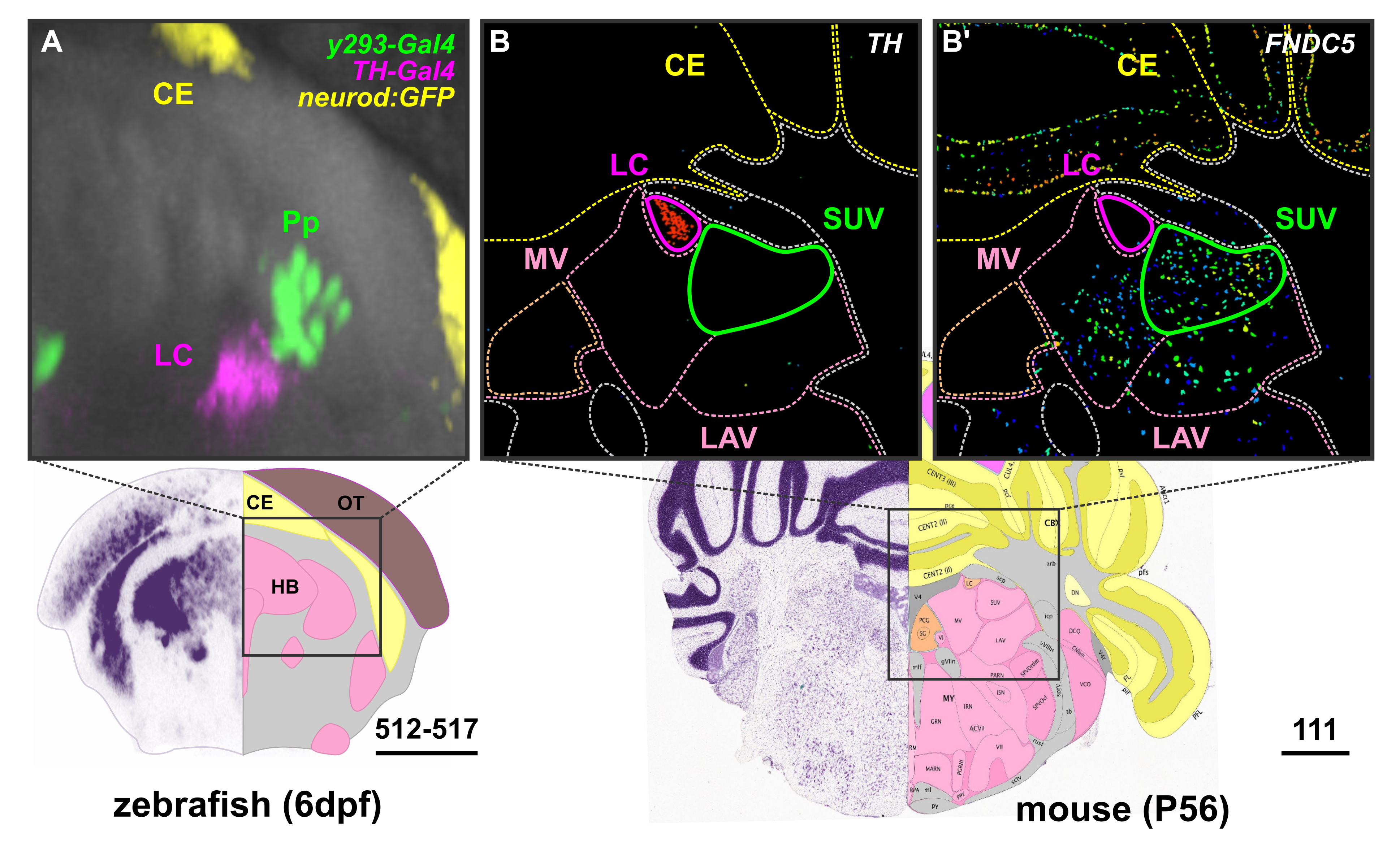

Supplement: S4 Fig — (A) Bottom: coronal projection through zebrafish rhombomere 1 (slice 512–517 from ZBB) with nuclear labeling on the left (elavl3:nls-mCar in purple) and neuroanatomic segmentation on the right (magenta, OT; yellow, CE; pink, medulla oblongata; gray, neuropil). Top: coronal projection of the outlined region showing y293-Gal4 (green, prepontine neurons), neurod:GFP (yellow, CE) [59], and TH-Gal4 (magenta, LC) [60]. (B) Bottom: mouse P56 coronal section (slice 111 from the AMBA) with Nissl-staining on the left (purple) and neuroanatomic segmentation on the right (magenta, superior colliculus; yellow, CE; pink, medulla oblongata; gray, fiber tracts). Top: AMBA in situ hybridization images for TH (B) and Fndc5 (B'). Image credit: Allen Institute, modified from Allen Developing Mouse Brain Atlas. AMBA, Allen Mouse Brain Atlas; CE, cerebellum; Fndc5, fibronectin type III domain containing 5; HB, hindbrain; LAV, lateral vestibular nucleus; LC, locus coeruleus; MV, medial vestibular nucleus; OT, optic tectum; P56, postnatal day 56; Pp, prepontine area; SUV, superior vestibular nucleus; TH, tyrosine hydroxylase; ZBB, Zebrafish Brain Browser. (TIF) [file pbio.3000480.s006.tif]

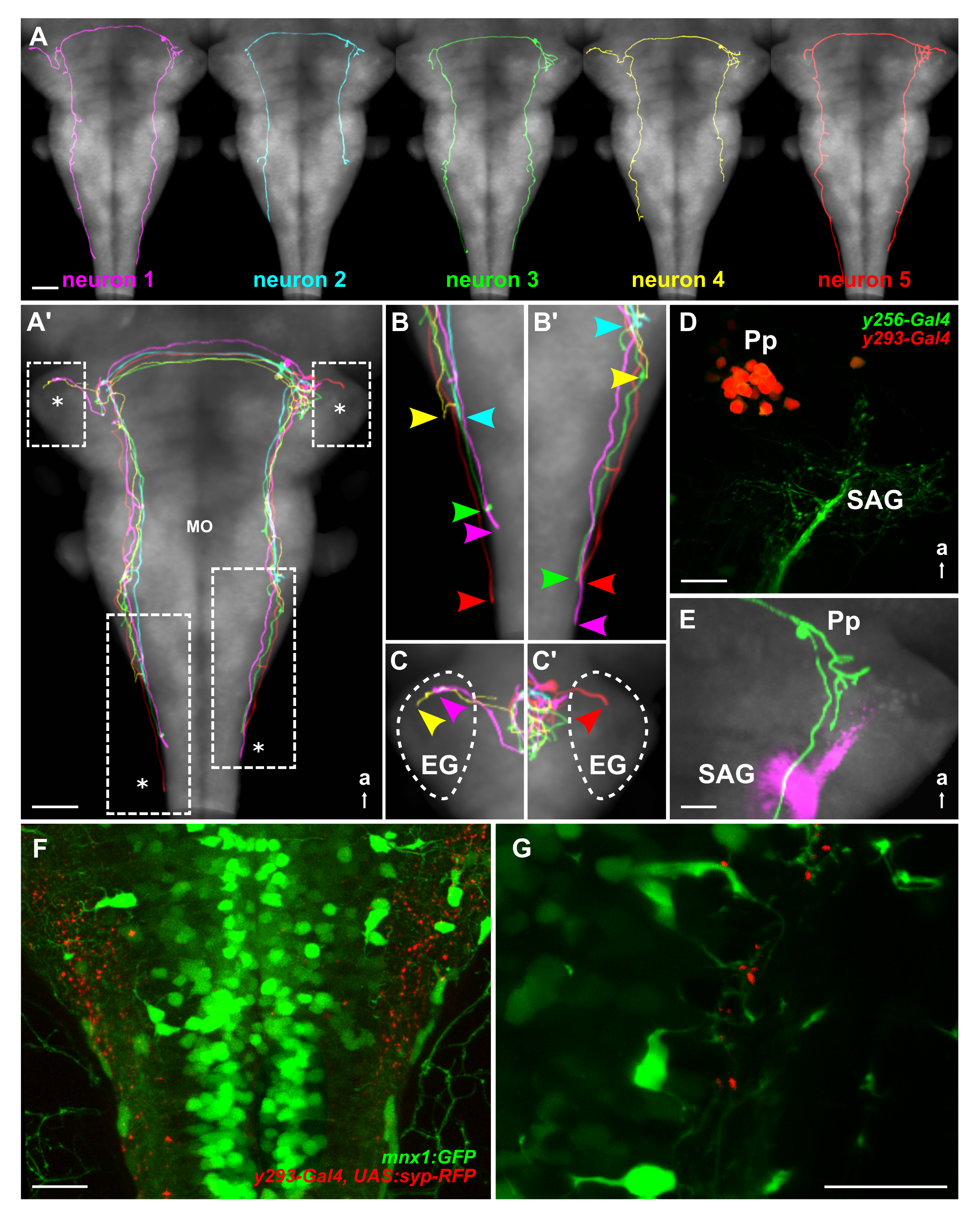

Supplement: S5 Fig — (A) Dorsal standard-deviation projections of 5 traced prepontine escape neurons with elavl3:Cer as a reference (gray). (A’) Overlay of co-registered neurons in (A) showing conserved quadripartite morphology (asterisks). Dotted lines indicate areas expanded in (B) and (C). (B) Enlargements of hindbrain from A’ with arrowheads indicating neuron terminals. (C) Enlargements of lateral rhombomere 1 from A’ with arrowheads marking termini in the cerebellar EG. (D) Horizontal projection of confocal stack including prepontine escape neuron cell bodies (“Pp,” y293-Gal4; UAS:KaedeR, red) after selective photoconversion of Kaede to red and SAG axon rostral termini (y256-Gal4;UAS:KaedeG, green). (E) Projection of a reconstructed neuron (green) registered to ZBB, with the y256-Gal4 pattern (magenta) that labels the SAG and its projections. (F-G) Horizontal projection (F) and zoom (G) of confocal stack through the caudal hindbrain and anterior spinal cord of a y293-Gal4, UAS:synaptophysin-TagRFPT, mnx1-GFP larva. Scale bars: 50 μm in (A) and (A'); 20 μm in (D-G). EG, eminentia granularis; SAG, statoacoustic ganglion; ZBB, Zebrafish Brain Browser. (TIF) [file pbio.3000480.s007.tif]

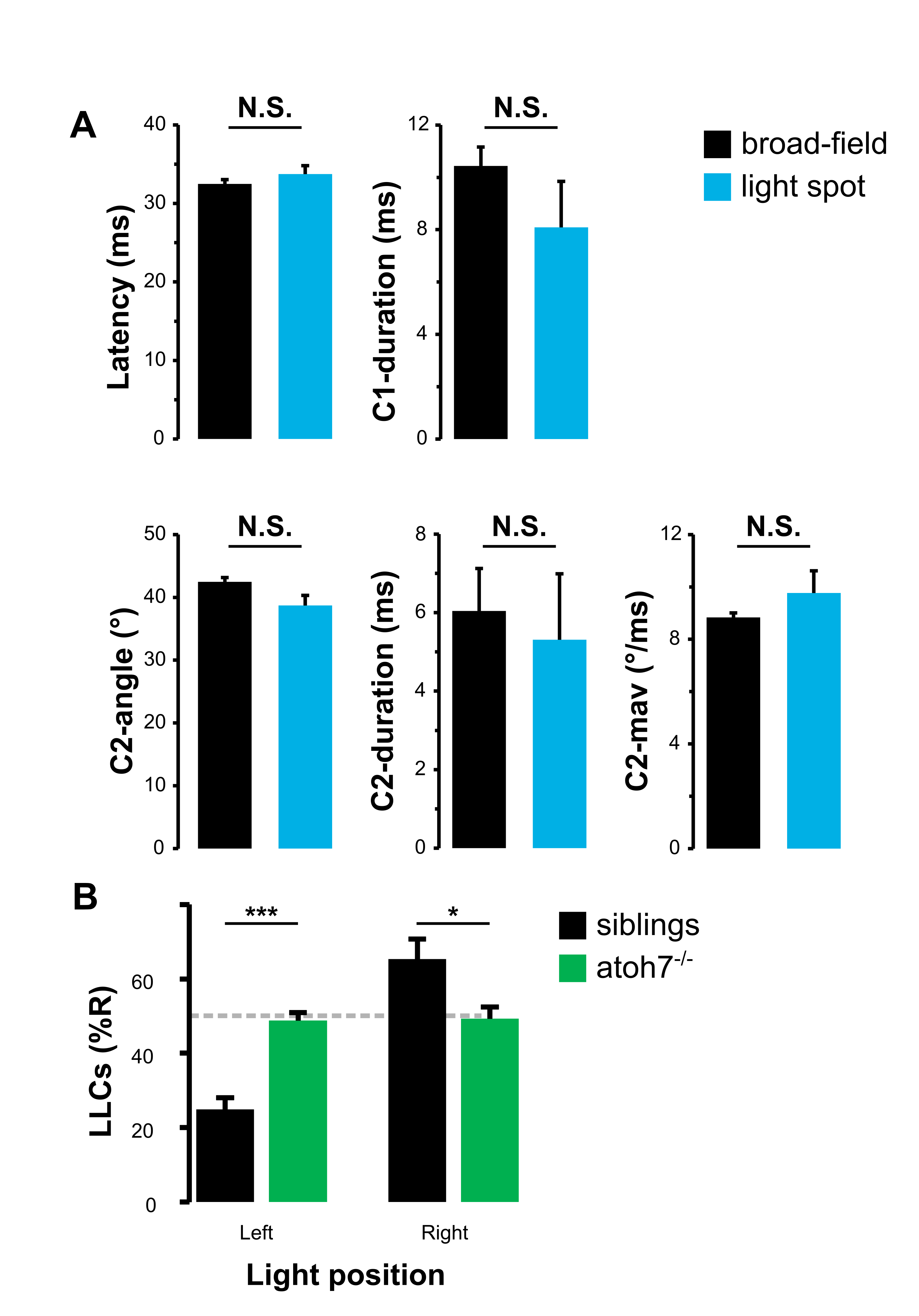

Supplement: S6 Fig — (A) Kinematic parameters for acoustically evoked LLCs performed under broad-field illumination (black) or in the presence of a light spot (blue). n = 29 groups of larvae. (B) Percent of LLCs in a rightward direction in the presence of a light spot for atoh7 mutant larvae and siblings. ***p < 0.001, *p < 0.05, n = 5 plates each atoh7−/− and siblings. Underlying numerical data are included in S2 Data. atoh7, atonal bHLH transcription factor 7; C1, initial C-start bend; C2, counterbend; LLC, long-latency C-start. (TIF) [file pbio.3000480.s008.tif]
